# Towards a Safe Pathway to Biological Parenthood for Pulmonary Arterial Hypertension

**DOI:** 10.1002/pul2.70325

**Published:** 2026-05-18

**Authors:** Jamie D. Ingram, Scott M. Nelson, Melanie J. Brewis, Martin K. Johnson, Colin Church

**Affiliations:** ^1^ Scottish Pulmonary Vascular Unit Golden Jubilee National Hospital Glasgow Scotland UK; ^2^ School of Medicine, Dentistry and Nursing University of Glasgow Glasgow Scotland UK

**Keywords:** fertilisation in vitro, ovarian hyperstimulation syndrome, pre‐implantation genetic testing, pulmonary hypertension, surrogacy

## Abstract

Pulmonary arterial hypertension (PAH) carries a high risk of maternal morbidity and mortality in pregnancy, and current guidelines advise against conception. For women with PAH who wish to have genetically related children, in vitro fertilisation (IVF) combined with gestational surrogacy presents a viable and increasingly safe alternative. Advances in IVF protocols—including gonadotropin‐releasing hormone (GnRH) antagonist stimulation, agonist trigger, personalised gonadotropin dosing, and elective embryo cryopreservation—have markedly reduced the risks of ovarian hyperstimulation syndrome and made oocyte retrieval feasible even in high‐risk populations. The addition of pre‐implantation genetic testing for monogenic disorders (PGT‐M) enables the selection of embryos free of heritable PAH‐related mutations, while pre‐implantation genetic testing for aneuploidy (PGT‐A) improves implantation rates and reduces miscarriage. For women who, due to personal, cultural or legal reasons, decline surrogacy and choose to carry a pregnancy themselves, a conservative approach—using natural cycle frozen embryo transfer and single euploid embryos—can reduce, but not eliminate, risk. The risks of pregnancy in this population are acknowledged to be high, and the guidelines continue to mandate against PAH patients becoming pregnant. However, there are alternative strategies developing which may allow biological parenthood without some of the inherent cardiopulmonary risk. This paper reviews the available evidence and proposes a clinical framework for offering IVF safely in this population, while advocating for the development of a dedicated registry and expert consensus to guide future care.

## 1 Background

Pulmonary arterial hypertension (PAH) in pregnant women has long been associated with alarmingly high maternal mortality rates. Despite advancements in medical therapies, multidisciplinary management during pregnancy and the peri‐partum period, maternal mortality remains significant, ranging from 11% to 25% [1, 2, 3, 4, 5]. Indeed, PAH is listed as an mWHO 2.0 class IV condition, recognising this high‐risk profile in pregnancy [6]. Consequently, the European Society of Cardiology (ESC) and the European Respiratory Society (ERS) guidelines for pulmonary hypertension (PH) continue to recommend that clinicians advise against pregnancy in women with PAH [7]. Despite these recommendations, many patients still express a desire for biological parenthood.

While adoption and various forms of surrogacy—including traditional surrogacy and donor egg surrogacy—offer alternatives that circumvent the maternal risks associated with PAH, some women prefer options that maintain a biological genetic link with their children. For these individuals, in vitro fertilisation (IVF) with gestational surrogacy, where the affected mother's eggs are used to create embryos for implantation in a surrogate, emerges as a potential pathway to parenthood. When combined with pre‐implantation genetic testing, exclusion of non‐viable embryos or those with PAH‐associated genetic mutations is possible.

The avoidance of planned pregnancy in women with PAH has resulted in a limited body of literature examining IVF in this context [8, 9]. Previous reviews on the subject have highlighted that both IVF and harvesting of eggs are not advised in PAH. Furthermore, serious worsening events can occur with ovarian hyperstimulation [6, 10]. However, these concerns do not reflect modern advances in IVF practices and a recent consensus statement for medically assisted reproduction in cardiovascular disease has suggested that IVF with a view to surrogacy could be feasible, even in mWHO class IV groups [11].

This paper will explore the feasibility, and address the associated challenges, of the concept of gestational surrogacy in patients with PAH, utilising current best obstetric practice. It will offer a framework to develop an international registry so that the PAH community can learn about this evolving technique.

### 1.1 In Vitro Fertilisation in Pulmonary Hypertension

A comprehensive literature review was conducted from 1946 to October 2025 using OVID Medline. Search terms included *pulmonary hypertension*, *pulmonary arterial hypertension*, *PAH*, *assisted reproductive technologies*, *assisted reproductive techniques*, *embryo transfer*, *fertility preservation*, *in vitro fertilisation*, *oocyte retrieval*, *ovulation induction*, *surrogate mothers*, *ovarian stimulation*, and *ovarian hyperstimulation syndrome* (OHSS). Only two case reports of patients with PH who underwent IVF were identified. To the author's knowledge, no additional PH‐specific case reports, systematic reviews, or guidelines exist on this topic in the published literature.

The first report, published in 2004, describes a 28‐year‐old nulliparous woman with idiopathic PAH, maintained on intravenous epoprostenol therapy (22 ng/kg/min), warfarin and digoxin [8]. Invasive haemodynamics showed mean pulmonary artery pressure of 71 mmHg and echocardiogram noted significant right ventricular (RV) dilatation and dysfunction. The woman underwent four IVF cycles using a healthy 23‐year‐old gestational carrier. The first cycle, following a low‐dose Gonadotropin‐releasing hormone (GnRH) agonist protocol, was cancelled due to poor ovarian response. In cycle two, ovarian stimulation with 300 IU recombinant follicle stimulating hormone (recFSH—a highly pure synthetic form of FSH) and 300 IU highly purified human menopausal gonadotropin (HPhMG) yielded eight mature oocytes. Intracytoplasmic sperm injection was performed, resulting in six embryos with three transferred to the carrier, but no pregnancy occurred.

For cycle three, a GnRH antagonist protocol replaced the GnRH agonist protocol, yielding eight oocytes and producing three embryos which were transferred concurrently, resulting in a successful singleton pregnancy in the gestational carrier. Before starting a teratogenic medication for her progressing PH, the patient underwent a fourth IVF cycle using the same protocol as the third, with seven embryos frozen at the pronuclear stage for future use. The patient tolerated general anaesthesia for all retrievals with no perioperative complications.

The report notes PH progression several months later but does not speculate on whether this was related to the IVF process [8]. It is well established that the RV is under increased strain during pregnancy, which can lead to progressive RV failure in patients with PAH [10]. However, there is no evidence to suggest that ovulation induction or oocyte retrieval directly impacts long‐term outcomes in PH. In this case, it is likely that the progression was unrelated to the IVF cycles.

The second report, published in 2012 [9], describes a 25‐year‐old woman with a complex medical history, including mixed connective tissue disease (MCTD), PH, interstitial lung disease, and antiphospholipid syndrome complicated by bilateral pulmonary embolism. She sought fertility preservation prior to undergoing high‐dose alkylating agent therapy and autologous bone marrow transplantation for her worsening pulmonary status. After extensive consultation, she opted for controlled ovarian stimulation and embryo cryopreservation, with plans to use a gestational carrier for future childbearing.

Her warfarin prescription was switched to enoxaparin, and to mitigate the risk of ovarian hyperstimulation syndrome (OHSS), a GnRH antagonist protocol was used for IVF, with 150 IU of recFSH and 75 IU of HPhMG. Final oocyte maturation was triggered with 10,000 IU of hCG. Sixteen oocytes were retrieved, six of which were fertilised and cryopreserved at the pronuclear stage.

Two days after retrieval, the patient was hospitalised with multifocal pneumonia and worsening PH. Her condition deteriorated into ventilator‐dependent respiratory failure, complicated by gastrointestinal bleeding, thrombosis, and ICU‐acquired myopathy. After a prolonged hospitalisation, she was discharged to an acute rehabilitation facility. Over the following year, she experienced a spontaneous pregnancy, which ended in miscarriage. Given the risks of pregnancy, she underwent permanent sterilisation and was reportedly considering transferring her frozen embryos to a gestational carrier.

These cases underscore some potential complications that may arise during oocyte retrieval in patients with PAH, while also highlighting outdated practices that would not be used in modern IVF. Reducing the risk of OHSS—characterised by vascular endothelial growth factor (VEGF)‐driven capillary leakage leading to third space losses, reduced cardiac preload, tachycardia, hypotension, and increased risk of thrombosis through haemoconcentration [12]—is a key target of modern protocols. These complications are challenging for any patient but could be catastrophic in the context of an impaired RV. Additionally, the effect of oestrogen—which increases during stimulation protocols—in PAH is not fully understood, though appears to have a paradoxical role of contributing to disease susceptibility while also supporting RV function [13, 14]. There have been a number of changes in the modern protocols. Today, GnRH antagonist protocols are favoured over agonist protocols, high doses of gonadotropins have been replaced with validated dosing algorithms incorporating pre‐treatment characteristics, hCG triggering is replaced by safer alternatives such as GnRH agonist triggers to reduce the risk of OHSS, general anaesthesia is typically avoided for oocyte retrieval in favour of conscious sedation, and the transfer of three embryos would not be recommended due to the associated risks of multiple pregnancies.

## 2 Suggested Ovarian Stimulation Protocol in Patients With PAH

**FIGURE 1:**
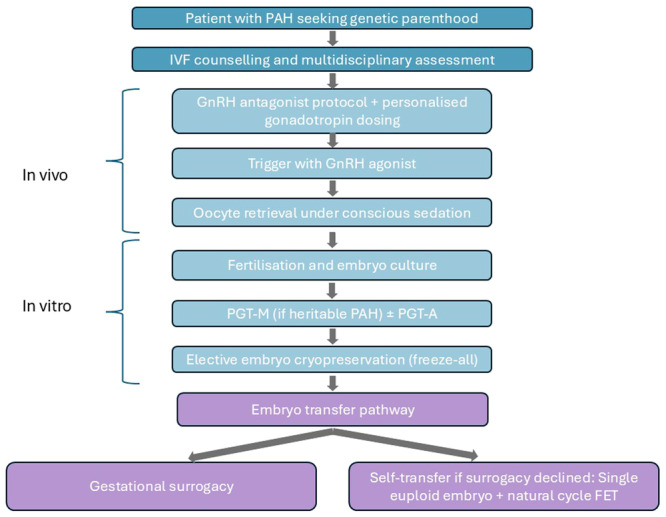
Flowchart of proposed IVF protocol for patients with PAH.

This section outlines recent advances in IVF protocols that, when combined, provide a significantly safer ovarian stimulation approach and could serve as a template for patients with PAH (see Figure 1).

### 2.1 Patient Selection

Patients considering IVF require case‐by‐case evaluation at a PAH MDT and in discussion with anaesthetics, high‐risk obstetrics and reproductive medicine specialists. Preferred candidates would be WHO functional class I–II, ESC/ERS low or intermediate‐low risk with stable disease, adequate RV function and ideally recent haemodynamic right heart catheterisation assessment. Discussion should emphasise that pregnancy in PAH remains discouraged and that ovarian stimulation and oocyte retrieval in PAH are low‐evidence areas. However, where patients remain intent on proceeding, their decision could be supported with PH optimisation, an optimised stimulation protocol and careful monitoring.

### 2.2 Gonadotropin‐Releasing Hormone (GnRH) Antagonist Protocols

A GnRH antagonist protocol is recommended for patients at risk of OHSS as it immediately blocks GnRH receptors in the pituitary gland, preventing premature release of luteinizing hormone (LH) and follicle‐stimulating hormone (FSH). This reduces the risk of excessive follicle development and allows more controlled ovarian stimulation. In contrast, GnRH agonist protocols cause an initial surge of LH and FSH which can increase the risk of OHSS. Moreover, antagonist cycles permit the use of a GnRH agonist trigger instead of human chorionic gonadotropin (hCG), further reducing the risk of OHSS, as hCG is a key driver of the condition by stimulating VEGF [15]. A Cochrane review reported that switching to GnRH antagonist protocols, even while maintaining an hCG trigger, was associated with a significantly lower incidence of OHSS, with an odds ratio of 0.43 (95% CI 0.33–0.57), representing a nearly 60% reduction in risk compared to GnRH agonist protocols [16].

### 2.3 Follicular Growth and Development – Choice of Gonadotropin

Exogenous administration of FSH and LH is a standard approach to stimulating ovarian follicles in IVF. HPhMG, which contains both FSH and LH activity, has been widely used for many years and is an effective option for reducing the risk of OHSS. The MEGASET and MEGASET HR trials demonstrated that HPhMG protocols, particularly in high responders, result in lower rates of OHSS compared to recFSH. In the MEGASET HR trial, OHSS occurred in 4.5% of patients treated with HPhMG vs. 7.6% with recFSH, with an odds ratio of 0.58 (95% CI 0.38–0.89), despite protocols primarily relying on hCG triggers and fresh embryo transfers—practices now recognised as risk factors for OHSS [17].

More recently, the use of follitropin delta, a recFSH, has introduced personalised dosing algorithms based on characteristics such as body weight and anti‐Müllerian hormone (AMH) levels. In a pivotal trial, the use of follitropin delta led to an OHSS incidence of 1.5%, compared to 4.3% with standard dosing, with an odds ratio of 0.35 (95% CI 0.17–0.69), demonstrating substantial reduction in OHSS risk while maintaining comparable live birth rates [18]. Across multiple trial populations, individualised follitropin delta dosing has consistently demonstrated reductions in both early OHSS and/or preventative interventions (adjusted OR 0.27, 95% CI 0.15, 0.49) and early moderate/severe OHSS (adjusted OR 0.30, 95% CI 0.16, 0.58) [19]. For PAH patients, for whom even mild OHSS could have serious cardiopulmonary consequences, this personalised individualised ovarian stimulation approach represents a significant advancement in making ovarian stimulation safer.

### 2.4 GnRH Agonist Trigger

The use of a GNRH agonist (GnRHa) trigger for final oocyte maturation has revolutionised the safety of IVF. Unlike hCG, which promotes sustained luteal activity and VEGF production—key drivers of OHSS—the GnRHa trigger induces a short, physiological surge of LH and FSH, effectively maturing oocytes while facilitating rapid luteolysis and minimising vascular permeability. Robust clinical data, including Cochrane reviews, consistently demonstrate a significant reduction in OHSS risk—by up to 85%—with GnRHa compared to hCG [20]. Importantly, in cycles where no embryo transfer is planned, such as for fertility preservation or egg donation, the physiological demands of the luteal phase are irrelevant. In this context, the risk of OHSS is virtually abolished, as there is no need for exogenous luteal support and no potential for early pregnancy‐related stimulation of the ovaries. Thus, when combined with a GnRH antagonist protocol, a GnRHa trigger followed by elective oocyte or embryo cryopreservation represents an exceptionally safe approach to IVF in women with PAH or others at risk of cardiopulmonary compromise, with no meaningful risk of OHSS or related complications.

### 2.5 Oocyte Retrieval

Oocyte retrieval is typically performed 36 h after administration of a GnRH agonist trigger, using transvaginal ultrasound‐guided aspiration and almost universally carried out under conscious sedation. General anaesthesia can be associated with significantly increased risk in patients with PAH, including perioperative RV failure, hypoxia, and fatal cardiovascular collapse [21]. However, in this protocol it is rarely required for oocyte retrieval. Conscious sedation—often using agents such as propofol, midazolam, and fentanyl—provides a safer alternative with a rapid recovery profile and is the preferred method of anaesthesia in IVF clinics globally [22]. Anaesthetic plans should be carefully individualised, with close monitoring and minimal sedation to reduce the risk of respiratory depression. Adjunctive techniques such as paracervical block may further reduce the need for systemic sedatives [22]. Given these considerations, oocyte retrieval under conscious sedation in a stable PAH patient can be performed safely in experienced IVF centres with appropriate precautions and multidisciplinary support.

### 2.6 Cryopreservation

Elective cryopreservation of all embryos—referred to as a “freeze‐all” strategy—has emerged as a pivotal advancement in assisted reproduction. This approach decouples the ovarian stimulation phase from embryo transfer, allowing the patient's hormonal milieu to normalise, particularly avoiding the supraphysiologic oestradiol levels that can compromise endometrial receptivity and exacerbate OHSS risk. Elective freezing reduces OHSS incidence and may improve outcomes in hyper‐responders without increasing miscarriage or perinatal complications [23, 24, 25, 26]. Furthermore, studies have suggested that frozen‐thawed embryo transfers (FET) may offer superior endometrial synchrony, more physiological implantation environments, and potentially lower risks of preterm birth and low birthweight compared to fresh transfers [27, 28]. While cryopreservation is not a medical requirement in gestational surrogacy—since the surrogate has not been exposed to ovarian stimulation—the freeze‐all strategy still offers clinical advantages. It facilitates embryo selection based on pre‐implantation genetic testing, allows flexible scheduling of transfer to optimise surrogate endometrial receptivity, and avoids the challenges of coordinating fresh transfers across two individuals.

### 2.7 Pre‐Implantation Genetic Testing

Pre‐implantation genetic testing for monogenic disorders (PGT‐M) allows for the identification and exclusion of embryos carrying disease‐causing mutations, while preserving genetic relatedness. Pathogenic variants in genes such as BMPR2, EIF2AK4, TBX4, and others have been implicated in inherited forms of PAH. Carriers of BMPR2 mutations, for instance, often present earlier and with more severe haemodynamic compromise than non‐carriers [29, 30]. In the context of gestational surrogacy for women with PAH—where pregnancy is contraindicated—PGT‐M provides an ethical and precise means to reduce future disease burden and offer a healthy start for the child.

For patients with PAH who do not carry a heritable mutation, or following PGT‐M exclusion of affected embryos, pre‐implantation genetic testing for aneuploidy (PGT‐A) can be employed to further refine embryo selection. PGT‐A identifies chromosomally normal (euploid) embryos, which are associated with significantly higher implantation rates and lower miscarriage rates compared to aneuploid embryos. Recent data demonstrate that cumulative live birth rates following transfer of euploid embryos exceed 90% by the third transfer and 98% by the fifth transfer, even in single embryo transfer strategies [31, 32]. In this context, PGT‐A complements PGT‐M by ensuring that only unaffected, viable embryos are transferred, thus maximising reproductive success and minimising risk.

### 2.8 IVF in PAH Patient Groups Who Do Not Wish to Pursue Gestational Surrogacy

Although gestational surrogacy offers the safest reproductive pathway for women with PAH, some individuals may decline this option for religious, cultural, legal, or personal reasons and instead express a strong desire to carry a pregnancy themselves. While pregnancy remains high risk in this population, IVF may still be considered in select cases as part of a carefully individualised, multidisciplinary care plan. In such scenarios, the goal shifts from eliminating to minimising maternal risk. The use of modern IVF protocols allows oocyte retrieval to be undertaken with a high degree of safety, even in women with cardiopulmonary compromise. Elective embryo cryopreservation enables cycle segmentation and defers transfer until a later date, offering time for further medical optimisation or reassessment.

If the patient elects to proceed with embryo transfer into her own uterus, a cautious and conservative approach is essential. The selection of a single, unaffected, euploid embryo—confirmed via PGT‐M and PGT‐A—minimises the risks associated with multiple pregnancy and chromosomal abnormalities. Employing a natural cycle frozen embryo transfer (NC‐FET) can further reduce pregnancy complications compared to artificial cycles. A systematic review and meta‐analysis demonstrated that NC‐FET is associated with a significantly lower risk of hypertensive disorders of pregnancy, including preeclampsia, compared to artificial cycle FET [33]. Additionally, NC‐FET has been linked to reduced incidences of placenta accreta, postpartum haemorrhage, and large‐for‐gestational‐age neonates [34]. Despite these precautions, pregnancy in PAH patients remains inherently hazardous. Therefore, such decisions necessitate extensive multidisciplinary counselling with clear documentation of informed consent and close monitoring throughout the pregnancy.

### 2.9 Medication Management During IVF

All pulmonary vasodilator therapy can be continued in freeze‐all cycles with gestational surrogacy; there is no direct teratogenic risk to the embryo, as it is never exposed to maternal medications during its development. However, teratogenic medications, including endothelin receptor antagonists (bosentan, ambrisentan, and macitentan) and riociguat should be discontinued prior to planned embryo self‐transfer to ensure clinical stability can be maintained without these agents. Phosphodiesterase‐5 inhibitors (sildenafil and tadalafil) and prostacyclin analogues should be continued throughout. Patients on warfarin require bridging to low‐molecular‐weight heparin (LMWH) peri‐retrieval. Prophylactic LMWH during stimulation is recommended for non‐anticoagulated patients due to the prothrombotic effects of elevated estradiol combined with baseline PAH‐associated thrombotic risk.

## 3 Conclusions

### 3.1 Limitations

Although an emerging area of interest and research, there is currently a limited body of evidence relating to IVF and surrogacy in patients with PAH. Therefore perspectives explored in this review reflect general advances in reproductive medicine which the authors believe are applicable to the PAH cohort.

### 3.2 Outlook

Despite these progresses, there remains a critical lack of formal clinical pathways, prospective outcome data, and guideline consensus for managing fertility care in this vulnerable population. We strongly advocate for the creation of national and international registries to capture IVF outcomes in PAH, and for reproductive health and cardiopulmonary societies to develop structured guidance through expert consensus.

### 3.3 Conclusion

For women with PAH, the desire for biological parenthood has long been constrained by the substantial risks posed by pregnancy. However, with a considered multidisciplinary approach and improving therapeutic options for PAH, evidence suggests that these risks may not be as high as they once were, particularly in patients with low‐risk disease [35, 36, 37, 38]. Therefore, as a community we should start to consider the possibility of pregnancy becoming more manageable in low‐risk cohorts of PAH following detailed individualised assessment and with specialist PH centre support. Advances in assisted reproductive technologies now offer a realistic and increasingly safe alternative through IVF, particularly when combined with gestational surrogacy.

## Author Contributions

All authors contributed to the manuscript. **Jamie D. Ingram:** conceptualisation, writing – original draft, literature search, figure preparation. **Scott M. Nelson:** writing – review and editing. **Melanie J. Brewis** and **Martin K. Johnson:** writing – review and editing. **Colin Church:** conceptualisation, writing – review and editing, supervision. All authors approved the final manuscript.

## Funding

The authors have nothing to report.

## Ethics Statement

The authors have nothing to report.

## Conflicts of Interest

Scott M. Nelson has participated in advisory boards and received consultancy or speaker fees from Access Fertility, Beckman Coulter, Ferring, Finox, Merck, Modern Fertility, MSD, Roche Diagnostics, and The Fertility Partnership. Colin Church, Jamie D. Ingram, Melanie J. Brewis, and Martin K. Johnson have no conflicting interests.

## Supporting information

Supporting File

## Data Availability

Data sharing is not applicable to this article, as no new data were created or analysed in this study.
