# Supplementary material for: Towards a Safe Pathway to Biological Parenthood for Pulmonary Arterial Hypertension
Source: Pulm Circ. 2026 May 18;16(2):e70325. doi: 10.1002/pul2.70325 (PMC13183777; doi:10.1002/pul2.70325)
Supplement: Supplementary file 1 — Supporting File [file PUL2-16-e70325-s001.docx]

Online Supplements

Appendix 1: Glossary and Definitions

| **Term** | **Definition** |
| --- | --- |
| **Traditional Surrogacy** | The surrogate’s own eggs are fertilised in vitro with the intended father’s sperm; the surrogate is both the genetic and gestational mother. |
| **Donor Egg Surrogacy** | An egg from a third-party donor is fertilised with the intended father’s sperm and implanted into a surrogate, who carries the pregnancy but has no genetic relationship to the child. |
| **Gestational Surrogacy** | The eggs of the affected mother are fertilised in vitro and implanted into a surrogate; the surrogate has no genetic link to the child. |
| **Fertility Preservation** | The process of freezing oocytes or embryos for future use, typically prior to medical treatment or in the context of high-risk conditions. |
| **Pre-implantation Genetic Testing for Monogenic Disorders (PGT-M)** | Genetic testing of embryos to identify and exclude those carrying specific inherited gene mutations associated with monogenic diseases, such as heritable PAH. |
| **Pre-implantation Genetic Testing for Aneuploidy (PGT-A)** | Screening of embryos for chromosomal abnormalities (e.g. trisomies or monosomies) to identify chromosomally normal (euploid) embryos with higher implantation and live birth potential. |
| **GnRH Antagonist Protocol** | An ovarian stimulation protocol used in IVF that prevents premature ovulation by directly and rapidly suppressing gonadotropin release from the pituitary. |
| **GnRH Agonist Trigger** | An alternative to hCG for triggering final oocyte maturation, which reduces the risk of ovarian hyperstimulation syndrome by inducing a physiological LH surge. |
| **Freeze-All Strategy** | An approach in which all embryos are cryopreserved after retrieval and no fresh transfer is performed, allowing for subsequent frozen embryo transfer in a hormonally optimal and safer cycle. |
| **Natural Cycle Frozen Embryo Transfer (NC-FET)** | A method of embryo transfer that aligns with the woman’s natural ovulatory cycle, avoiding the use of exogenous hormones and reducing risks associated with artificial endometrial preparation. |
| **Ovarian Hyperstimulation Syndrome (OHSS)** | A potentially serious complication of ovarian stimulation characterised by enlarged ovaries and fluid shifts due to increased vascular permeability, historically triggered by hCG. |
| **Euploid Embryo** | An embryo with a normal number of chromosomes, associated with a higher likelihood of implantation and lower risk of miscarriage or chromosomal disorders. |
| **mWHO 2.0** | Modified World Health Organisation classification system for maternal cardiovascular risk in pregnancy |
